# Clustering single-cell multi-omics data via multi-subspace contrastive learning with structural smoothness

**DOI:** 10.1093/bib/bbag005

**Published:** 2026-01-27

**Authors:** Yun Ding, Yangzhen Jiang, Jing Wang, Dayu Tan, Yansen Su, Chunhou Zheng

**Affiliations:** School of Artificial Intelligence, Anhui University, 111 Jiulong Road, Hefei 230601, China; School of Artificial Intelligence, Anhui University, 111 Jiulong Road, Hefei 230601, China; School of Artificial Intelligence, Anhui University, 111 Jiulong Road, Hefei 230601, China; Institute of Physical Science and Information Technology, Anhui University, 111 Jiulong Road, Hefei 230601, China; School of Internet, Anhui University, 3 Feixi Road, Hefei 230601, China; School of Artificial Intelligence, Anhui University, 111 Jiulong Road, Hefei 230601, China

**Keywords:** single-cell, multi-omics clustering, contrastive learning, graph neural network

## Abstract

The integration of single-cell multi-omics data can uncover the underlying regulatory basis of diverse cell types and states. However, single-cell data inherently suffer from high levels of noise, sparsity, and intercellular heterogeneity, which pose significant challenges to the accuracy and robustness of clustering algorithms. Most existing multi-omics clustering approaches primarily focus on the integration of omics individuality and commonality across modalities, but they ignore the diverse feature extraction of the low-dimensional representation before the fusion of single-cell multi-omics data, and the feature smoothing consistency of the diverse features after the fusion of single-cell multi-omics data. In order to address above issues, we propose a novel multi-subspace contrastive learning with structural smoothness method for single-cell multi-omics data clustering (scMUSCLE), which is designed to address the challenges inherent in multi-omics data integration. First, the proposed scMUSCLE method leverages the degree structure to enhance structural diversity of each omics modality. Second, we perform multi-subspace contrastive learning to improve the diversity exploration across multi-omics features. Next, we propose an adaptive graph convolution clustering module, which establishes an adaptive feedback mechanism between intra-cluster smoothness and the downstream clustering task. Extensive experiments on four benchmark multi-omics datasets demonstrate the effectiveness and robustness. The source code can be found on the GitHub repository: https://github.com/GodIsGad/scMUSCLE.

## Introduction

Single-cell multi-omics joint sequencing technologies, such as sci-CAR [1, 2], SNARE-seq [3], Paired-seq [4], and 10$\times $ Genomics Multiome [5, 6], have seen rapid development. These sequencing methods perform simultaneous sequencing of gene expression and chromatin accessibility within the same cell, providing valuable data resources for the development of single-cell multi-omics clustering methods. Therefore, how to effectively integrate single-cell multi-omics data remains a formidable challenge due to its inherent high sparsity [7–9], substantial heterogeneity caused by measurement noise [10], the large dimensionality difference between scATAC-seq and scRNA-seq data, $\sim $10–20 times [11, 12], and the imbalance of information between omics [13, 14], all of which pose significant computational and analytical challenges. The integration of single-cell multi-omics data can facilitate the study of complex biological information [15, 16], and researchers have been developing single-cell multi-omics integration methods using machine learning and bioinformatics techniques [17, 18]. Clustering analysis is an essential step in most single-cell studies and has been studied extensively. Clustering techniques of single-cell multi-omics data can be broadly categorized into two types of approaches: matrix factorization-based methods and deep learning-based methods. Matrix factorization-based approaches are typically built upon techniques such as non-negative matrix factorization or principal component analysis, aiming at integrating multi-omics data and address cellular heterogeneity [19–21]. However, they are often sensitive to noise when handling multi-omics data, damaging the robustness of clustering results.

With the development of deep learning and its ability to automatically extract expressive features, deep learning methods have become the mainstream technology for single-cell data analysis. scEMC [22] takes into account the impact of the imbalanced information richness of different omics data, using the Transformer structure to learn global structural relationships in different feature spaces. scMVAE [23] provides a multimodal variational autoencoder for integrating scRNA-seq and scATAC data, which include three learning strategies to infer the distribution of multimodal cell data. Deep cross-omics cycle attention (DCCA) [24] utilizes separated deep generative networks to characterize the scRNA-seq and scATAC-seq data. Subtype-DCC [25] proposes the decoupled contrastive clustering method to learn clustering-friendly representations identifying cancer subtypes. scCobra [26] designs a deep generative neural network to mitigate batch effects, minimizes over-correction, and ensures biologically meaningful data integration without assuming specific gene expression distributions. Besides, scIMTA [27] is designed to enhance interpretability and effectively address the issues of topological structure preservation and dropout events. Meanwhile, Transformer-based or generative models such as scHiMe [28] and Smmit [29] are employed for predicting/imputing methylation signals or achieving robust integration across samples and modalities. Most of them focus on a shared representation, but disregard the omics individuality, scMCs [30] is to simultaneously consider both the individuality and commonality of omics information, which integrates individual modality-specific features and shared cross-omics features. In addition, graph convolutional networks (GCNs) [31, 32] are well suited for modeling intercellular relationships and are highly scalable, making them ideal for large-scale single-cell multi-omics datasets. For example, the weighted Nearest Neighbor method [33] was proposed to connect similar cells based on consensus information from two modalities [34]. GCN-sc [35] utilizes a mutual nearest-neighbor algorithm to identify pairs of units with the closest Euclidean distance and then constructs a hybrid graph based on relationships between units. scMFG [13] combines omics feature groups and graph structure decomposition to uniformly represent hierarchical structural information, effectively identifying rare subpopulations, and improving clustering accuracy on real single-cell multi-omics data.

**Figure 1.**
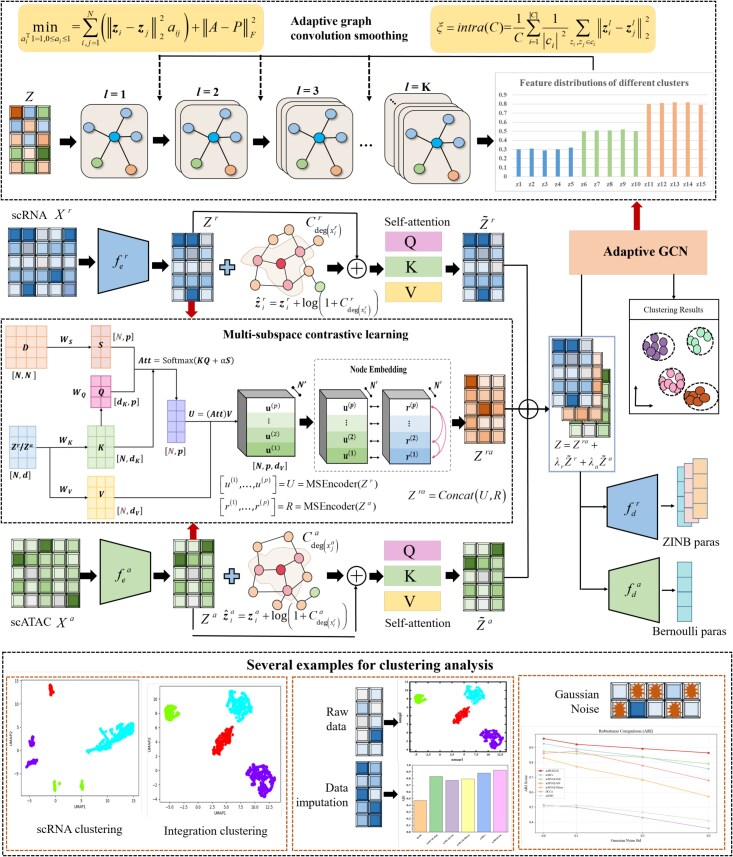
An Overview of the scMUSCLE framework for single-cell multi-omics clustering. The scMUSCLE framework first projects the scRNA-seq data $ X^{r} $ and scATAC data $ X^{a} $ into different low-dimensional spaces $ Z^{r} $ and $ Z^{a} $ using autoencoders specific to each omics type. Second, we leverages the degree structure to obtain enhanced structural feature representation $ \hat{Z}^{r}$ and $ \hat{Z}^{a}$ of each omics modality. Subsequently, we employs self-attention mechanisms to capture the omics-specific features $ \tilde{Z}^{r} $ and $ \tilde{Z}^{a} $. Next, we propose the multi-subspace contrastive learning to explore more comprehensive diverse features $ Z^{ra} $ across multiple subspaces. Afterward, we integrate the omics-specific and common information to obtain the common embedding matrix $ Z $ and learns the parameter representations of the zero-inflated negative binomial (ZINB) distribution $ \{\Pi , \bar{M}_{r}, \Theta \} $ and the Bernoulli distribution $ \bar{M}_{a} $. Finally, we propose to establishing an adaptive feedback mechanism between intra-cluster smoothness of integrated diverse features and the downstream clustering task based on fused features $ Z $, thereby enhancing its clustering capabilities.

Although the aforementioned methods are effective, they still have some limitations. First, most of them focus on the extraction of common features between the single-cell multi-omics data or the extraction of individual features of each omics data. However, they ignore the extraction of effective diversity features of the low-dimensional representation before the fusion of single-cell multi-omics data, leading to an inability to obtain distinctive feature representations of biological features for data imputation and cell clustering. Second, most methods only perform the downstream tasks after the fusion of single-cell multi-omics features, and do not take into account the inconsistency of fused diverse features, leading to the imperfect adaptation of downstream tasks. In other words, they ignore the feature smooth consistency after the fusion of single-cell multi-omics characteristics, leading to suboptimal clustering performance. In summary, the key contributions are as follows. (i) We propose the multi-subspace contrastive learning module extracting shared cross-omics features while preserving the feature diversity across multiple latent subspaces. (ii) We design the centrality encoding mechanism enhancing structural diversity within individual omics via degree-structured representations. (iii) We proposed the adaptive graph convolutional neural network by establishing an adaptive feedback mechanism between intra-cluster smoothness and the downstream clustering task. (iv) Extensive validations on four benchmark datasets confirm superior clustering accuracy and robustness. The subspace concept refers to a set of representations of different multi-omics data within same embedding space. Note that when the number of multi-subspace is set to one, the multi-subspace contrastive learning reduces to single space contrastive learning. In order to address these challenges, we propose the scMUSCLE method. The overview of the scMUSCLE framework is shown in Fig. 1.

## Materials and methods

### Structural diversity enhancement

As a classical neural network, the autoencoder can map high-dimensional data to a low-dimensional representation space. Therefore, we employ autoencoders to map single-cell multi-omics data into their respective nonlinear embedding spaces, preserving individual features, and enhancing robustness against noise and outliers [36].

Let $X^{r}\in \mathbb{R}^{N\times Dr}$, $X^{a}\in \mathbb{R}^{N\times Da}$ be the normalized scRNA-seq data matrix and scATAC-seq data matrix, respectively, where *N* denotes the number of samples, and $X^{r}$ and $X^{a}$ denote the feature matrix. The scMUSCLE framework initially employs two independent encoders $ f_{e}^{r} $ and $ f_{e}^{a} $ to learn the corresponding $ d $-dimensional feature representations $ \{Z^{r}, Z^{a}\} \in \mathbb{R}^{N \times d} $,


(1) \begin{align*}&Z^{r}=f_{e}^{r}(X^{r}),\quad Z^{a}=f_{e}^{a}(X^{a}),\end{align*}

where $ d $ denotes the dimension of the embedding space, $ Z^{r} $ denotes the latent low-dimensional representation between cells and genes in the scRNA-seq data, and $ Z^{a} $ denotes the latent pattern between cells and peaks in the scATAC data.

The quality of the low-dimensional features has been improved. But the diversity of low-dimensional feature is limited. We propose to incorporate the structural information of the graph into the individual omics information, enhancing the diversity of the low-dimensional feature of each omics information. Specific details are as follows.

We firstly adopt an adaptive probability graph method to construct the graph structure between cells. Because the high-dimensional sparsity and noise between single cells often have a bad influence on the establishment of graph structure, leading to poor quality of graph structure, this adaptive probability graph can assign a soft probability value to the similarity between cells instead of assigning a hard value of 0 or 1 to the similarity between cells, thus avoiding the influence of high-dimensional sparsity and noise between cells to some extent. The mathematical expression of the adaptive probability graph is denoted as,


(2) \begin{align*}& \min_{a_{i}^{T}1=1,0\leq a_{i}\leq1}\sum_{i,j=1}^{N}\left(\left\|z^{r}_{i}-z^{r}_{j}\right\|_{2}^{2}\right)a_{ij})+\left\|A-P\right\|_{F}^{2},\end{align*}

where $ P \in \mathbb{R}^{N \times N} $ denotes the guidance matrix obtained by the radial basis function, $ A \in \mathbb{R}^{N \times N} $ denotes the adaptive probability matrix, and $ N $ denotes the total number of nodes. $ a_{ij} \in \mathbb{R}^{1 \times N} $ denotes the vector representing the similarity between cell $ i $ and cell $ j $, while $ z^{r}_{i} $ and $ z^{r}_{j} $ are the feature information of cell $ i $ and cell $ j $ in scRNA-seq data, respectively. Similarly, we can also obtain the adaptive probability graph for the low-dimensional representation of scATAC data. We propose a structural centrality encoding that assigns a real-valued embedding vector to each node based on its degree. Since centrality encoding is applied to each node, it can be simply added to the node features as,


(3) \begin{align*}&\hat{z}^{r}_{i}=z^{r}_{i}+\log{(1+C^{r}_{{\mathrm{deg}}(x^{r}_{i})})},\hat{z}^{a}_{i}=z^{a}_{i}+\log{(1+C^{a}_{{\mathrm{deg}}(x^{a}_{j})})},\end{align*}

where $ \hat{Z}^{r} = [\hat{z}^{r}_{1}, \hat{z}^{r}_{2}, \dots , \hat{z}^{r}_{N}] \in \mathbb{R}^{N \times d} $, $ \hat{Z}^{a} = [\hat{z}^{a}_{1}, \hat{z}^{a}_{2}, \dots , \hat{z}^{a}_{N}] \in \mathbb{R}^{N \times d} $, and $ \{C^{r}, C^{a}\} \in \mathbb{R}^{d} $ are learnable embedding vectors specified by the degree structures $deg(x^{r}_{i})$ and $deg(x^{a}_{j})$, and the degree structure can be obtained by $ deg(x^{r}_{i}) $= $ \sum _{j=1}^{N}{a_{ij}} $. Similarly, the degree structure of $deg(x^{a}_{j})$ is also obtained by adaptive probability graph method via Equation (2). The purpose of using addition here is to enhance the structural diversity of the original features, and the purpose of using log function is to smooth the newly added structural features as much as possible for the subsequent downstream clustering tasks. In order to better integrate these two features, the self-attention mechanism is adopted, and the normalized attention score matrices are defined as,


(4) \begin{align*}&\mathrm{A_{tt}^{r}}=\mathrm{softmax}\left(\frac{\hat{Z}^{r}(\hat{Z}^{r})^{T}}{\sqrt{d}}\right),\mathrm{A_{tt}^{a}}=\mathrm{softmax}\left(\frac{\hat{Z}^{a}(\hat{Z}^{a})^{T}}{\sqrt{d}}\right).\end{align*}

Then, in order to better adapt the original features and new structural features, a low-dimensional representation in the cell is constructed, and the following semantic fusion is carried out. The mathematical expression is written as follows:


(5) \begin{align*}&\tilde{Z}^{\mathrm{r}}=\mathrm{A}_{\mathrm{tt}}^{\mathrm{r}}\hat{Z}^{r},\tilde{Z}^{\mathrm{a}}=\mathrm{A}_{\mathrm{tt}}^{\mathrm{a}}\hat{Z}^{a}.\end{align*}

Through the above process, the individual features extracted by the autoencoders for each specific omics modality are significantly enhanced, and it not only combines the structural information of the graph, but also combines the global context awareness, improving the diversity of feature representations.

### Multi-subspace contrastive learning

Next, we conduct multi-subspace contrastive learning among multi-omics features, fully exploiting the feature diversity of multi-omics based on common characteristics. The specific details are as follows.

Firstly, we define two views $ u $ and $ r $, where $ u $ denotes the low-dimensional embedding $ Z^{r} \in \mathbb{R}^{N \times d} $, and $ r $ denotes the low-dimensional embedding $ Z^{a} \in \mathbb{R}^{N \times d} $. However, unlike the data augmentation in traditional contrastive learning, the views in this study are considered from the perspective of features. We perform multiple subspace encoders (MSEncoder) to generate representations of multiple subspaces from the $ u $-view and $ r $-view. This process can be expressed as,


(6) \begin{align*} &\begin{bmatrix} \mathrm{u}^{(1)},...,\mathrm{u}^{(\mathrm{p})} \end{bmatrix}=\mathrm{U}=\mathrm{MSEncoder}\left(Z^{r}\right), \end{align*}

(7) \begin{align*} &\left[\mathrm{r}^{(1)},\ldots,\mathrm{r}^{(\mathrm{p})}\right]=\mathrm{R}=\mathrm{MSEncoder}(Z^{a}), \end{align*}

where $ p $ denotes the number of subspaces, $ \{u^{(i)}, r^{(i)}\} \in \mathbb{R}^{d/p} $, $ u^{(i)} $ and $ r^{(i)} $ are the node embeddings of the $ i $th subspace, and $ d $ represents the feature dimension.

Next, the MSEncoder can be regarded as a multi-feature generator based on the self-attention mechanisms. It processes the input feature matrix $ Z^{r} $ through two distinct linear projections, thereby mapping it into key embeddings $ K $ and value embeddings $ V $.


(8) \begin{align*}& K = Z^{r} W_{K} \in \mathbb{R}^{N \times d_{K}}, \quad V = Z^{r} W_{V} \in \mathbb{R}^{N \times d_{V}},\end{align*}

where $ W_{K} \in \mathbb{R}^{N \times d_{K}} $ and $ W_{V} \in \mathbb{R}^{N \times d_{V}} $ denote the different projection matrices corresponding to keys, values. To focus on the distinct subspaces within node features, we introduce a set of query vectors $ Q = [q_{1}, q_{2}, \dots , q_{p}] \in \mathbb{R}^{d_{K} \times p} $, where each query vector represents a subspace and guides a specific representation. The number of query vectors determines the number of target subspaces. Similar operations with Equation (8) for $ Z^{a} $.

In order to obtain the diversity features captured by multi-subspace contrastive learning and focus on more critical node information, the degree information obtained from the graph structure is further utilized. We proposed to integrate graph structure information into the attention mechanism, thereby enhancing the discriminative power of diversity feature representations. The degree embedding $ S $ is initialized as follows:


(9) \begin{align*}& S = D W_{S} \in \mathbb{R}^{N \times p},\end{align*}

where $ D \in \mathbb{R}^{N \times N} $ denotes the degree structure information obtained by Equation (2), and $ W_{S} \in \mathbb{R}^{N \times p} $ denotes the projection matrix corresponding to the degree. We apply structural information to the attention mechanism to enhance its ability to capture structural diversity. The specific mathematical expression is as follows:


(10) \begin{align*}& \mathrm{Att} = \mathrm{Softmax}(K Q + \alpha S).\end{align*}

The integration between the attention mechanism and the graph structure enhances structural awareness and feature robustness in single-cell information extraction, promoting structural alignment of cross-modal features. Next, we calculate the node embeddings within each subspace, broadcasting $ V $ to each subspace to obtain $ V_{p} \in \mathbb{R}^{N \times p \times d_{V}} $. Ultimately, we achieve the node embeddings for $ p $ subspaces, which can be formulated as,


(11) \begin{align*}&\left[\mathrm{u}^{(1)},...,\mathrm{u}^{(\mathrm{p})}\right]=\mathrm{U}=(Att)\mathrm{V}_{p}\in\mathrm{R}^{N\times p\times d_{V}}.\end{align*}

We then compute $[u^{(1)}, \dots , u^{(p)}]$ and $[r^{(1)}, \dots , r^{(p)}]$ by maximizing the mutual information (MI) between node representations across subspaces and minimizing the MI within subspaces, which forms the basis of their subspace contrastive loss function.

Specific multi-subspace contrastive loss function used are introduced as follows. The representations of the outputs from $ p $ subspaces for the two views $ u $-view and $ r $-view are given by $ \{(u^{(k)}, r^{(k)}) | k = 1, 2, \dots , p\} $. Our goal is to optimize the MSEncoder so that $ U $ and $ R $ have diversified feature representations. To achieve this goal, we use a pair of loss functions that focus on maximizing the MI between subspace representations and minimizing the MI between different subspace representations. For the MI between subspace representations, we can formulate the objective as:


(12) \begin{align*}& \max \sum_{\theta,\phi}^{p} \mathrm{MI}(u^{(k)}, r^{(k)}) ,\end{align*}

where $ \theta $ and $ \phi $ denote the parameters of the encoders in the $ u $-view and $ r $-view branches, respectively. However, since $ u^{(k)} $ and $ r^{(k)} $ are unknown, the MI cannot be computed directly, making the estimation of MI a crucial issue. A common alternative to maximizing MI is to use sampling-based methods to maximize its lower bound estimate [37, 38]. Typically, the Jensen–Shannon (JS) estimation is used in place of the Kullback–Leibler estimation, with the formula given as follows:


(13) \begin{align*}& \begin{split} \mathrm{MI}_{JS}(\mathbf{u}^{(k)}, \mathbf{r}^{(k)}) = &\mathbb{E}_{P}(\mathbf{u}^{(k)},\mathbf{r}^{(k)})[-SP(-\mathcal{D}(\mathbf{u}^{(k)},\mathbf{r}^{(k)}) )]- \\&\mathbb{E}_{P}(\mathbf{u}^{(k)})\mathbb{E}_{P}(\mathbf{r}^{(k)})[SP(\mathcal{D}(\mathbf{u}^{(k)},\mathbf{r}^{(k)})], \end{split}\end{align*}

where $ \mathrm{SP}(x) = \log (1 + e^{x}) $ and $ D(\cdot , \cdot ): \mathbb{R}^{d} \times \mathbb{R}^{d} \rightarrow \mathbb{R} $ serves as a neural network that accepts representations from the two views as inputs and evaluates the degree of their consistency. The discriminator is directly designed to compute the dot product of the two views $D\left (u_{i}^{(k)},r_{i}^{(k)}\right )=\left (u_{i}^{(k)}\right )^{T}r_{i}^{(k)}$.

In addition to the objectives within each subspace, constraints are imposed on the relationships between different subspaces within the same view to increase the diversity of the subspaces. It is worth noting that we have proposed an optimization objective to minimize MI as much as possible. This objective encourages any representation pair within view $ u $ to capture various aspects of the given graph. For $ \{(u^{(k)}), (u^{(l)}) | 1 \leq k < l \leq p\} $, the expression can be written as,


(14) \begin{align*}& \min_{\theta} \frac{1}{2} \sum_{k=1}^{p} \sum_{l=1}^{p} \mathrm{MI}(u^{(k)}, u^{(l)}).\end{align*}

In order to minimize the MI, an upper bound of MI as an efficient estimation. The contrastive log-ratio upper bound (CLUB) is adopted here,


(15) \begin{align*}&I_{\mathrm{CLUB}}=\mathbb{E}_{P(x,y)}[logP(y|x)]-\mathbb{E}_{(P(x))}\mathbb{E}_{(P(y))}[logP(y|x)].\end{align*}

The key to computing CLUB is estimating the distribution $ P(y|x) $. Here, we assume that each dimension of $ x $ and $ y $ is related, which gives us the hypothesis $ E(y|x) = x $, meaning each dimension of $ y $ depends only on the corresponding dimension of $ x $. Substituting $ u^{(k)} $ and $ u^{(l)} $ for $ x $ and $ y $, respectively, the distribution of $ u^{(l)} $, given $ u^{(k)} $, is determined as follows:


(16) \begin{align*}& \begin{aligned} P\left(u^{(l)} \mid u^{(k)}, \Sigma\right) & = \mathcal{N}\left(u^{(l)} \mid u^{(k)}, \beta^{-1} I\right) \\ & = \prod_{i} \mathcal{N}\left(u_{i}^{(l)} \mid u_{i}^{(k)}, \beta^{-1}\right), \end{aligned}\end{align*}

where $ \beta ^{-1} $ denotes the variance shared across all dimensions, and $ u_{i}^{(l)} $ and $ u_{i}^{(k)} $ denote the $ i $-th dimensions of $ u^{(l)} $ and $ u^{(k)} $, respectively.

The final objective function of the subspace contrastive learning module is the weighted sum of the above MI maximization and minimization objectives, which can be expressed by the following formula:


(17) \begin{align*}& \mathcal{L}_{\mathrm{contra}} = \max \sum_{\theta,\phi}^{p} \mathrm{MI}\left(r^{(k)}, u^{(k)}\right) - \gamma \sum_{k=1}^{p} \sum_{l=k+1}^{p} \mathrm{MI}\left(u^{(k)}, u^{(l)}\right),\end{align*}

where $ \gamma $ denotes the parameter that balances the influence of within-space and between-space objectives. Its core idea is to simultaneously achieve “correlation maximization” and “redundancy minimization” in the multi-omics feature space. Moreover, this formula ensures that the learned multi-omics features preserve shared biological consistency while preserving structural differences and omics-specific characteristics in feature space. Specifically, the objective function consists of two components. To integrate the learned representations from multiple views, we concatenate the trained embeddings $ U $ and $ R $ to obtain the unified common embedding $ Z^{ra} \in \mathbb{R}^{N \times d} $:


(18) \begin{align*}& Z^{ra} = \mathrm{Concat}(U, R).\end{align*}

### Multi-omics data fusion

Based on the above diversity features obtained by structural enhancement and multi-subspace contrastive learning, the next step is to effectively fuse these extracted features. In this process, we introduce scaling parameters $ \lambda _{r} $ and $ \lambda _{a} $ to perform an element-wise weighted summation of these two sets of latent representations, thereby achieving their effective aggregation. Through this approach, we can generate a more discriminative embedding representation $ Z $,


(19) \begin{align*}&Z=Z^{ra}+\lambda_{r}\tilde{Z}^{\mathrm{r}}+\lambda_{a}\tilde{Z}^{\mathrm{a}}\end{align*}

In the optimization of the embedding representation for multi-omics data, a common strategy is to use multiple multilayer perceptron decoders to reconstruct the data for each omics. However, frequent dropout events in the data can significantly affect the quality of the embedding representation $ Z $, thereby leading to biased clustering results. In order to tackle this challenge, we can impute the occurrence of dropout events and utilize these imputation results to adjust $ Z $, thereby improving the accuracy of key features. Studies have shown that scRNA-seq data often exhibit characteristics such as discreteness, variance greater than the mean, and high sparsity [39]. Despite this, some research indicates that the ZINB distribution can effectively describe these data characteristics [40, 41]. Therefore, the decoder network based on the ZINB model is used to explore the global probabilistic structure of scRNA-seq data. The ZINB distribution is mathematically defined by the mean $ \mu _{x} $ of the negative binomial distribution, the dispersion parameter $ \theta $, and the coefficient $ \pi $ that describes the probability of dropout events [42],


(20) \begin{align*} &\mathrm{NB}(x^{r};u_{r},\theta)=\frac{\Gamma(x^{r}+\theta)}{\Gamma(\theta)}\left(\frac{\theta}{\theta+u_{r}}\right)^\theta\left(\frac{u_{r}}{\theta+u_{r}}\right)^{x^{r}}. \end{align*}

(21) \begin{align*} &{\mathrm{ZINB}}(x^{r};\pi,u_{r},\theta)=\pi\xi_{0}(x^{r})+(1-\Pi)\mathrm{NB}(x^{r};u_{r},\theta). \end{align*}

Specifically, the ZINB-based decoder estimates the parameters $ \{\pi , \mu _{r}, \theta \} $ based on $ Z $ through three separate fully connected layers,


(22) \begin{align*} &\Pi=sigmoid\left(f_{d}^{r}(Z,W_\pi)\right)\qquad\qquad\qquad\qquad\quad \end{align*}

(23) \begin{align*} &\overline{\mathrm{M_{r}}}=\exp\left(f_{d}^{r}\left(Z,W_{u_{r}}\right)\right),\theta=\exp\left(f_{d}^{r}\left(Z,W_\theta\right)\right) \end{align*}

where $ \{\Pi , \bar{M}_{r}, \Theta \} $ denote the matrix forms of $ \{\pi , \mu _{r}, \theta \} $, and $ f_{d}^{r} $ is the decoder with fully connected layers; $ W_\pi $, $ W_{\mu _{r}} $, and $ W_\theta $ are three learnable parameter matrices. The activation function for $ \Pi $ is sigmoid, as the dropout probability lies between 0 and 1. Additionally, since the mean and dispersion parameters are non-negative, the exponential function $ \exp $ is chosen as the activation function for $ \bar{M}_{r} $ and $ \Theta $.

Unlike traditional autoencoders based on mean squared error loss, the loss function of the ZINB-based decoder network is the negative log-likelihood of the ZINB distribution,


(24) \begin{align*}&\mathrm{Ber}(x^{a};u_{a})=x^{a}log(u_{a})+(1-x^{a})\log(1-u_{a}),\end{align*}

where $ x^{a} $ denotes a vector from the original scATAC data, and $ u_{a} $ is the mean parameter of the Bernoulli distribution. The Bernoulli-based decoder estimates $ u_{a} $ based on $ Z $ through a fully connected layer with the sigmoid() function as the activation function,


(25) \begin{align*}&\overline{\mathrm{M_{a}}}=\mathrm{sigmoid}\left(f_{d}^{a}(Z,W_{u_{a}})\right),\end{align*}

where $ \bar{M}_{a} $ denotes the matrix form of $ u_{a} $, and $ W_{u_{a}} $ denotes the weight parameter matrix. Finally, the Bernoulli-based auto-decoder can be optimized through cross-entropy loss:


(26) \begin{align*}&\mathcal{L}_{\mathrm{Ber}}=\mathrm{CE}(X^{a},\overline{\mathrm{M}_{\mathrm{a}}}).\end{align*}

In pursuit of a more discriminative and informative common embedding representation that integrates the individual and common characteristics of multi-omics data, we unify the input targets for scRNA-seq data and scATAC data. The overall loss is formulated as follows:


(27) \begin{align*}& \begin{aligned} \mathcal{L}_{1} &= \operatorname*{argmin}_{\phi_{1}} \Big( -\log \left( \mathrm{ZINB}\left(X^{r} \mid \Pi, \overline{M_{r}}, \theta\right) \right) + \alpha_{1} \mathrm{CE}\left(X^{a}, \overline{M_{a}}\right) \\ &\quad + \alpha_{2} \Big( \max \sum_{\theta,\phi}^{p} \mathrm{MI}\left(r^{(k)}, u^{(k)}\right) - \gamma \sum_{k=1}^{p} \sum_{l=k+1}^{p} \mathrm{MI}\left(u^{(k)}, u^{(l)}\right) \Big) \end{aligned}\end{align*}

where $ \phi _{1} $ denotes the network parameters, and $ \alpha _{1} $ and $ \alpha _{2} $ are scalar parameters that constrain $ L_{\mathrm{Ber}} $ and $ L_{\mathrm{contra}} $, respectively. By optimizing the equation, the diversity features from individual and shared feature representations can be effectively fused from multi-omics data, and its quality of feature representation can be improved via the ZINB loss.

### Adaptive graph convolutional neural networks

In single-cell multi-omics clustering tasks, the smoothness of single-cell features is critical for downstream clustering task [43–45]. However, existing methods ignore the intrinsic smoothed feature information, we address this gap by establishing an adaptive feedback mechanism between intra-cluster smoothness of integrated diverse features and the downstream clustering task. Specifically, we first construct a graph structure from the fused features of single-cell multi-omics data based on previous adaptive probability graph in Equation (2). Subsequently, we apply GCN method to perform structural smoothness of graph convolution operations, facilitating feature information propagation via its massage-passing mechanism.

When we use the GCN model to further integrate omics information, the ReLU function is chosen as the activation function for the convolutional layers. The inputs to the GCN include the omics co-embedding matrix $ Z \in \mathbb{R}^{N \times d} $ and the adjacency matrix $ A_{f} \in \mathbb{R}^{N \times N} $, it can be obtained by,


(28) \begin{align*}&\min_{a_{fi}^{T}1=1,0\leq a_{fi}\leq1}\sum_{i,j=1}^{N}\left(\left\|z_{i}-z_{j}\right\|_{2}^{2}\right)a_{fij})+\left\|A_{f}-P\right\|_{F}^{2}.\end{align*}

When $ a_{fij} \ne 0 $, it means that cell $ i $ and cell $ j $ are each other’s neighbors with effective edge weights. When $ a_{ij} = 0 $, it means that cell $ i $ and cell $ j $ are not neighbors, and the diagonal elements of matrix $ A_{f} $ are set to zero to avoid the trivial solution. Since setting the diagonal elements to zero ignores the characteristics of the cells themselves, it is necessary to add self-loops to obtain the matrix $ A^{*} \in \mathbb{R}^{N \times N} $ in GCN. From matrix $ A^{*} $, the degree matrix $ D \in \mathbb{R}^{N \times N} $ is derived. $ D $ is a diagonal matrix, and to prevent the neighbor information of a cell from having an excessive influence on its features, we normalize matrix $ A^{*} $ using the degree matrix to obtain the normalized adjacency matrix $ \tilde{A} $, which is calculated as follows:


(29) \begin{align*}&\widetilde{\mathrm{A}}=\mathrm{D}^{-\frac{1}{2}}\mathrm{A}^{*}\mathrm{D}^{\frac{1}{2}}=\mathrm{D}^{-\frac{1}{2}}(\mathrm{A_{f}}+\mathrm{I})\mathrm{D}^{\frac{1}{2}},\end{align*}

where $ A^{*} = A_{f} + I $, and $ I \in \mathbb{R}^{N \times N} $ denotes the identity matrix.

The output representation of each layer of the GCN is denoted as $ X^{(l+1)} $, where,


(30) \begin{align*}&X^{l+1}=F\left(X^{l},\tilde{A}\right)=\sigma\left(D^{-\frac{1}{2}}A^{*}D^{\frac{1}{2}}X^{l}W^{l}\right).\end{align*}

In the model, we use Mean Squared Error as the loss function to measure the difference between the model’s output and the true labels. Specifically, assuming the model’s output is $ \hat{y}_{i} $ and the true data is $ y_{i} $, the loss function $ L_{2} $ is defined as:


(31) \begin{align*}&\mathcal{L}_{2}=\frac{1}{N}\sum_{i=1}^{n}\left(\widehat{y}_{i}-y_{i}\right)^{2}\end{align*}

where $ N $ is the number of samples, $ \hat{y}_{i} $ represents the model’s predicted output for the $ i $th sample, and $ y_{i} $ represents the true value of the $ i $th sample.

Next, in order to achieve the effective smoothing of fused diverse features in multi-omics data, it is necessary to determine the adaptive number of graph convolution layers $ k $ to avoid over-smoothing and under-smoothing phenomenon. Most existing methods determine the number of graph convolution layers by manually adjusting parameters, while we determine the optimal number of graph convolution layers based on the essence of feature smoothing of clusters. Specifically, we design an adaptive feedback threshold $ \xi $ based on the smoothness effect of fused omics feature information to determine the optimal number of graph convolution layers. In order to dynamically determine graph convolution layers, the intra-cluster performance measure $ \mathrm{intra}(C) $ [46, 47] is employed to assist in constructing the adaptive threshold. The adjustment of the number of iterations $ k $ (corresponding to $ k $ layers) can be guided by the clustering measure. The specific formula can be defined as follows:


(32) \begin{align*}&\xi=intra(C)=\frac{1}{|C|}\sum_{i=1}^{|C|}\frac{1}{|c_{i}|^{2}}\sum_{x_{i},x_{j}\in c_{i}}\left\|\widehat{x}_{i}-\widehat{x}_{j}\right\|_{2}^{2},\end{align*}

where $ C $ denotes the set of categories, $ |C| $ denotes the number of categories, $ c_{i} $ represents category $ i $, and $ |c_{i}| $ denotes the number of samples in category $ i $. The measure intra($ C $) represents the similarity among samples within each category. The formula is used to quantify the intra-cluster compactness of fused representations and serve as an adaptive threshold, and this metric evaluates feature smoothness by calculating the average squared Euclidean distance between sample representations within each cluster. A smaller $ \xi $ indicates closer proximity among samples, smoother representations, and higher intra-cluster consistency. Since graph convolution inherently performs Laplacian smoothing, too few convolutional layers result in insufficient feature fusion (under-smoothing), while excessive layers may induce over-smoothing, causing features across clusters to become indistinguishable and compromising discriminative power. Furthermore, the reason for using intra-cluster distance as the measurement criterion is as follows. GCN is essentially a form of Laplacian smoothing, which tends to make the features of adjacent samples more similar, thereby reducing the distance between samples. As $ k $ increases, the features of different omics become smoother, which can significantly reduce the intra-cluster distance and achieve the effect of optimizing the clustering task. Intuitively, different multi-omics datasets require a different number of convolutional layers to achieve effective smoothing and thereby optimize the fusion of different omics information. $ \mathrm{intra}(C) $ can effectively measure the smoothness of omics features. Omics datasets with sparse features may require more convolutional layers, while those with rich features may require fewer convolutional layers to achieve effective feature smoothing. Therefore, intra-cluster distance is used to evaluate the iterative effect.

We set $ \xi = \mathrm{intra}(C) $ as the early stopping condition for the iterations of the GCN. The adaptive iteration strategy is as follows: For each epoch, we calculate the intra-cluster distance for the current iteration and denote the intra-cluster distances at iteration $ t $ and iteration $ t + 1 $ as $ \xi ^{(t)} $ and $ \xi ^{(t+1)} $, respectively. If $ \xi ^{(t+1)} < \xi ^{(t)} $, we consider the features to be reasonably similar and proceed to the next iteration. If $ \xi ^{(t+1)}> \xi ^{(t)} $, we stop the iteration process at this point and record the current iteration layer $ t $. Stopping the iteration at the first local minimum prevents the occurrence of over-smoothing and ensures the effectiveness of the training process.

## Experiment results

### Cell clustering and visualization

**Figure 2.**
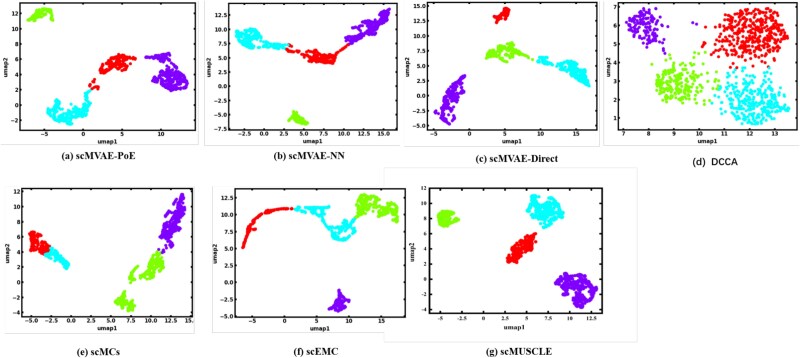
Cell clustering visualization of different methods on CellMix. (a) scMVAE-PoE, (b) scMVAE-NN, (c) scMVAE-Direct, (d) DCCA, (e) scMCs, (f) scEMC, and (g) scMUSCLE.

The details with datasets and evaluations can be found in supplementary data. Table 1 in supplementary data provides a comprehensive summary of the clustering performance of the scMUSCLE algorithm compared with other baseline methods across the four datasets. Each method was run independently five times, and the final results presented are the averages of these five runs. The entries highlighted in bold represent the methods that achieved the best performance on the corresponding metrics. The data in the table show that the scMUSCLE algorithm performed well on all four datasets in terms of the two key clustering metrics: NMI and ARI. To evaluate the quality of the embedding representation $ \hat{z} $, we employed Uniform Manifold Approximation and Projection (UMAP) [48–50] to visualize the clustering results of scMUSCLE and other baseline methods on each benchmark dataset. As shown in Fig. 2, different colors represent different clusters in the cell clustering and the clustering results of scMUSCLE exhibit the clearest separation boundaries and the more compact feature distribution.

**Table 1.** Performance of single clustering of compared methods on different datasets

		scMVAE-PoE	scMVAE-NN	scMVAE-Direct	DCCA	scMCs	scEMC	scMUSCLE
D1	NMI	0.8585	0.8308	0.8266	0.6190	0.8912	0.5740	**0.9313**
	ARI	0.8756	0.8611	0.8312	0.5134	0.9256	0.5033	**0.9594**
D2	NMI	0.3623	0.3538	0.2502	0.2634	0.3705	0.1446	**0.4084**
	ARI	0.3010	0.2948	0.2268	0.3114	0.3016	0.1060	**0.3363**
D3	NMI	0.5326	0.5294	0.4951	0.3102	0.5187	0.4258	**0.5635**
	ARI	0.3934	0.4043	0.3428	0.2415	0.3236	0.2756	**0.4338**
D4	NMI	0.5192	0.4922	0.5085	0.3485	0.4161	0.2553	**0.5373**
	ARI	0.3114	0.2945	0.2640	0.2501	0.2325	0.1784	**0.3442**

Generally, the proposed scMUSCLE method enhances inter-class segregation performance on the CellMix dataset, which is attributed to the integration of multi-subspace contrastive learning constraint, and adaptive graph convolution module. The proposed framework sustains simultaneous balance between intra-omics diversity and cross-omics coherence while substantially augmenting the discriminability and robustness of the integrated feature space. The clearly delineated class separation in Fig. 2 thus naturally reflects this design philosophy, additionally validating the scMUSCLE method’s efficacy and superiority in single-cell multi-omics feature processing and integration. More details with the visualization of cell clustering can be found in Supplementary Figs S1–S4.

### Evaluation of data imputation

In addition to accurate cell clustering, scMUSCLE also implements data imputation based on $ Z $ using two independent deep generative decoder networks with ZINB and Bernoulli parameters. To evaluate the quality of the imputed scRNA-seq and scATAC data, we visualized the original and imputed data generated by scMUSCLE, scMVAE-PoE, scMVAE-Direct, scMVAE-NN, and scMCs. Specifically, we projected the original and imputed data into different 2D spaces using UMAP and examined the cell clustering within them. We also used NMI and ARI to assess the clustering results provided by each method.

**Figure 3:**
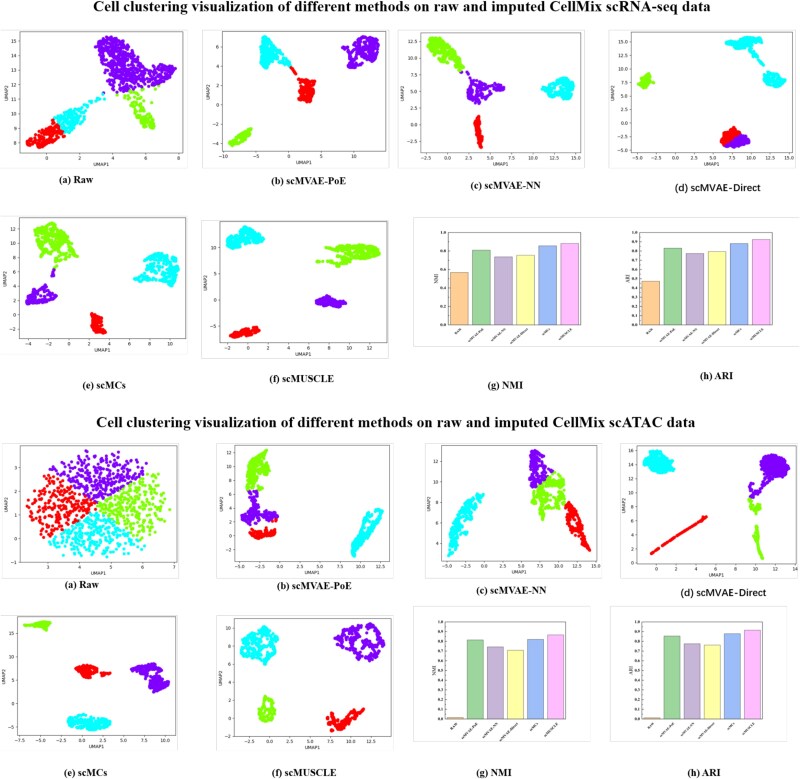
Cell clustering visualization of different methods on raw and imputed CellMix scRNA-seq and scATAC data. (a) scMVAE-PoE, (b) scMVAE-NN, (c) scMVAE-Direct, (d) DCCA, (e) scMCs, (f) scEMC, and (g) scMUSCLE.

Figure 3 reports the visualization and clustering performance of each method on the original and imputed data for CellMix datasets. We observe that the NMI and ARI scores of scMUSCLE are significantly higher than those of other baselines. The visualization results also confirm that the cell clusters identified by scMUSCLE are more separated between different clusters and more compact within clusters. All these findings confirm that scMUSCLE can generate informative embedding representations $ Z $ that can be used for data imputation. More details with sci-CAR, Peripheral Blood Mononuclear Cell (PBMC), and h3k4me3 datasets can be found in Supplementary Figs S5–S12.

### Performance comparison between single-omics and multi-omics clustering

**Figure 4:**
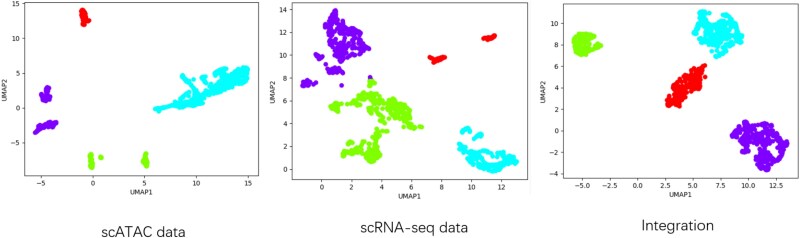
Cell clustering visualizations for scATAC data, scRNA data, and the integration of these two omics on the CellMix dataset.

At the single-cell level, multi-omics sequencing technologies can characterize cell states from different biological perspectives, providing more comprehensive cellular feature information. To this end, we designed experiments to compare single-omics clustering and multi-omics integration clustering. Specific experiments on CellMix are shown in Fig. 4. As shown in Fig. 4, for the clustering results of single scATAC and scRNA-seq data, the cells in the same cluster are significantly separated, and the clustering results are not compact, which seriously affects the subsequent cell annotation. However, the proposed scMUSCLE method is able to integrate multi-omics data and closely aggregate the cells in the same cluster together. More clustering results can be observed in Supplementary Figs S14–S18.

Therefore, we aim to achieve more accurate and stable cell clustering results by jointly modeling different omics data and fully exploring the complementary information between omics.

### Ablation study and parameter sensitivity analysis

**Figure 5:**
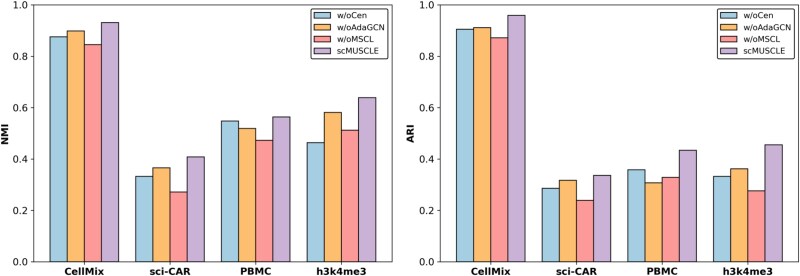
The performance of scMUSCLE and its variants.

To investigate the contributions of different components in scMUSCLE, we introduced three variants: w/oCen, w/oAdaGCN, and w/oMSCL, which, respectively, omit centrality encoding, adaptive graph convolutional neural network, and multi-subspace contrastive learning. Figure 5 displays the mean NMI and ARI values of scMUSCLE and its variants on four datasets. We observed that scMUSCLE significantly outperformed its variants, confirming that the centrality encoding, adaptive GCN, and multi-subspace contrastive learning modules indeed contribute to the quality of cell clustering. The main parameters include the parameters $ \alpha _{1} $ and $ \alpha _{2} $ of the loss function for multi-subspace contrastive learning, as well as the number of multi-subspaces $ \ p $. The experimental results on four datasets show that the loss caused by cross-entropy is relatively large, usually requiring a larger penalty, i.e. the control parameter $ \alpha _{1} $ will be smaller. After optimization through multi-subspace contrastive learning, the loss is smaller, and multi-group feature fusion can be better performed, usually requiring a moderate penalty parameter, i.e. the control parameter $ \alpha _{2} $ will be slightly larger. When the number of multi-subspaces $ \ p $ is set to 3, the experimental results on most datasets are better. More details can be found in Supplementary Figs S19–S22.

### Robustness evaluation under Gaussian noise perturbation

To verify the robustness of the proposed method scMUSCLE under data perturbation conditions, we designed a set of comparative experiments based on Gaussian noise interference. Specifically, we added Gaussian noise with a mean of 0 and standard deviations of 0.1, 0.3, and 0.5, respectively, to the original single-cell multi-omics data to simulate the measurement errors or biological variations that may occur during the actual data collection and processing. In this experiment, several representative comparison methods were selected, including scMCs, scMVAE-PoE, scMVAE-NN, scMVAE-Direct, DCCA, and scEMC. The performance of each method in the clustering task was evaluated, respectively, under different noise levels. NMI and ARI are adopted as evaluation indicators. The experimental results are shown in Fig. 7. The results show that despite the increase in noise intensity, scMUSCLE consistently maintains high clustering performance, demonstrating stronger robustness and stability.

**Figure 6:**
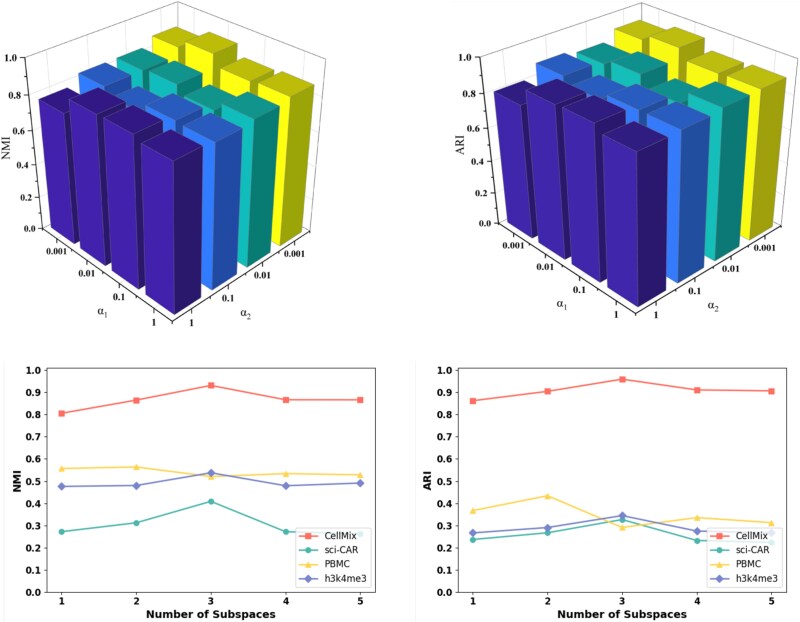
Sensitivity analysis of the parameters on scMUSCLE.

**Figure 7:**
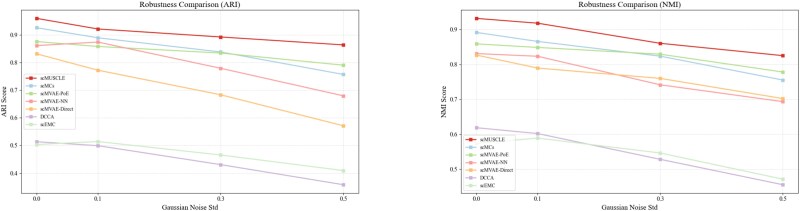
Robustness evaluation under Gaussian noise perturbation on the CellMix Dataset.

## Discussion and conclusion

We propose the scMUSCLE method, which is designed to address the challenges with the diverse feature before the fusion and the feature smoothing consistency of the diverse features after the fusion of single-cell multi-omics data. The proposed scMUSCLE mainly consists of structural feature diversity, multi-subspace contrastive learning as well as adaptive graph convolution smoothing. Extensive experiments including cell clustering, data imputation, ablation study, and robustness to noise on four benchmark multi-omics datasets demonstrate that the proposed scMUSCLE model outperforms other methods.

The main limitation of the scMUSCLE method lies in its multi-omics integration process, where the number of multi-subspaces remains manually specified. Unlike the adaptive feedback graph convolution module, which automatically determines the effective number of convolution layers, the lack of self-adjusting selection for subspace count may cause clustering performance to degrade sharply as noise intensity increases. A key direction for future research is to adaptively determine the number of subspaces based on single-cell heterogeneity mechanisms. This will enable the scMUSCLE method to effectively integrate diverse features with intra-cluster smoothness during training, preserving both single-cell heterogeneity and structural smoothness, thereby further enhancing clustering performance.

**Key Points**

- The single-cell multi-omics data clustering (scMUSCLE) aims at performing the diverse feature extraction of the low-dimensional representation, and the feature smoothing consistency of the diverse features.
- The scMUSCLE mainly consists of degree structure, multi-subspace contrastive learning as well as adaptive graph convolution smoothing modules.
- Compared with several clustering methods, the scMUSCLE achieves best clustering results on four single-cell multi-omics data.
- The scMUSCLE has stronger robustness on Gaussian noise and achieves best clustering results on raw and imputed scRNA-seq and scATAC data.

## Supplementary Material

scMUSCLE-supplementary_materials_bbag005

## Data Availability

The source code in this study is publicly available, and accessible online at https://github.com/GodIsGad/scMUSCLE.
