# Supplementary material for: Clustering single-cell multi-omics data via multi-subspace contrastive learning with structural smoothness
Source: Brief Bioinform. 2026 Jan 27;27(1):bbag005. doi: 10.1093/bib/bbag005 (PMC12834668; doi:10.1093/bib/bbag005)
Supplement: scMUSCLE-supplementary_materials_bbag005 [file scmuscle-supplementary_materials_bbag005.pdf]

scMUSCLE: a multi-subspace contrastive Learning framework for single-cell multi-omics data clustering

1 Datasets and Evaluation

scMUSCLE is a flexible framework that can integrate different types of single-cell omics data. In our experiments, we primarily evaluated the performance of scMUSCLE by jointly modeling scRNA-seq and scATAC data. We collected four preprocessed single-cell multi-omics datasets from other studies. We downloaded CellMix (D1, GSE 126074) from GEO, which contains 1047 cells with both chromatin accessibility and gene expression measured simultaneously in each single cell using SNARE-seq. The CellMix dataset contains a total of 1047 cells, including 4 cell types. The dimension of scRNA features is 501, and the dimension of scATAC features is 7137. We also collected the sci-CAR (D2) and PBMC (D3) datasets from the Con-AAE work. The sci-CAR dataset consists of a total of 1791 cells, including 3 cell types. The dimension of scRNA feature is 2614, and the dimension of scATAC feature is 916. The PBMC dataset includes 1927 cells with 19 distinct cell types. The dimension of scRNA feature is 51, and the dimension of scATAC feature is also 51. Additionally, we obtained the h3k4me3 (D4) dataset from the JSNMF work, which comprises 2684 cells with 20 distinct cell types. The dimension of scRNA feature is 5000, and the dimension of scATAC feature is 9903. The Adjusted Rand Index (ARI) and Normalized Mutual Information (NMI) are as evaluation metrics to evaluate the quality of the clustering results. To comprehensively evaluate the clustering performance, we conducted experiments on four multimodal single-cell datasets to compared with six competitive methods including scEMC, scMCs, scMVAE-PoE, scMVAE-NN, scMVAE-Direct, DCCA. More details with compared methods can be found in supplementary materials.

2 Performance of baselines on CellMix, sci-CAR, PBMC, and h3k4me3

In order to comprehensively evaluate the clustering performance of our scMUSCLEG, we conducted experiments on four multimodal single-cell datasets in this work, along with six competitive methods. scEMC introduced the SAN module, which combines a Transformer structure to capture global structural relationships in different feature spaces. scMCs employed multiple autoencoders to map omics data from high-dimensional spaces to low-dimensional spaces for analysis. scMVAE proposed three strategies—scMVAE-PoE, scMVAE-NN, and scMVAE-Direct—for learning joint latent features for data fusion and clustering. Among them, scMVAE-Direct directly connects the raw features of each omics, scMVAE-NN integrates low-dimensional features extracted from different omics, and scMVAE-PoE estimates the joint posterior distribution through a product of experts model. Additionally, DCCA projects different omics into corresponding low-dimensional spaces and utilizes a "teacher-student" mechanism to achieve effective fusion of multi-omics data.

We present the clustering results of the baselines on the CellMix, sci-CAR, PBMC, and h3k4me3 datasets in Figures S1 to S4. Figures S5 to S8 provide the visualization and clustering results of different methods on the original and imputed scRNA-seq data for each dataset, while Figures S9 to S12 display the visualization and clustering results for the original and imputed scATAC data. Based on these results and the content in the main text, we can observe that in most cases, the scMUSCLE method achieves clearer visualization results compared to the baseline methods. Additionally, the clustering results of scMUSCLE on the true values of CellMix, sci-CAR, PBMC, and h3k4me3 demonstrate that the scMUSCLE method

achieves the best imputation and clustering performance among the compared methods.

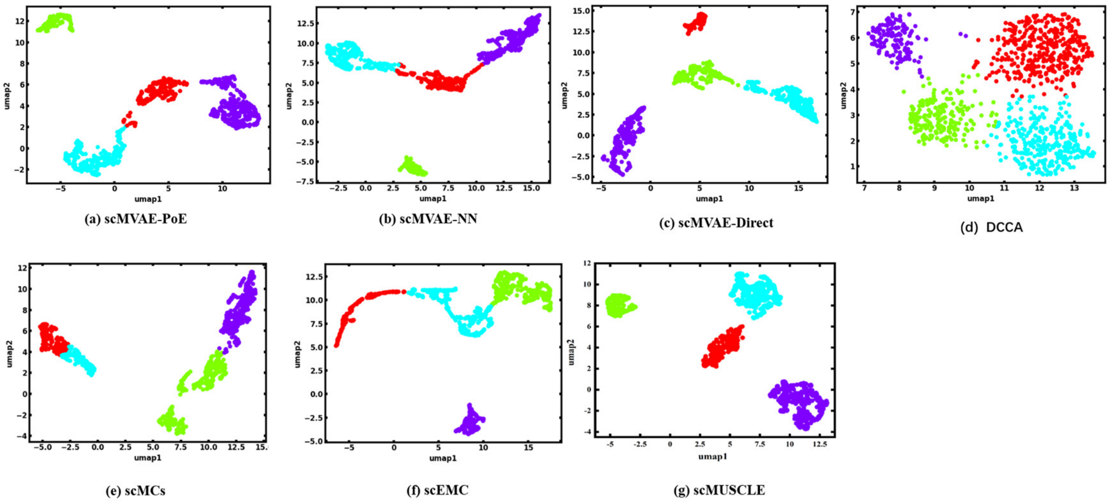

Figure S1: Cell clustering visualization of each method on CellMix. (a) scMVAE-PoE; (b)scMVAE-NN; (c) scMVAE-Direct; (d) DCCA; (e) scMCs; (f) scEMC; (g) scMUSCLE.

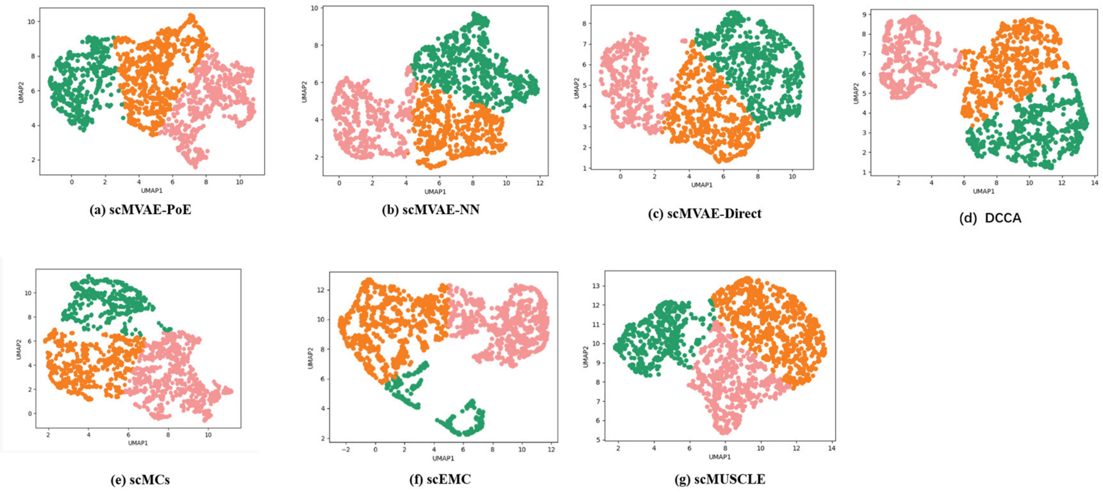

Figure S2: Cell clustering visualization of each method on sci-CAR. (a) scMVAE-PoE; (b)scMVAE-NN; (c) scMVAE-Direct; (d) DCCA; (e) scMCs; (f) scEMC; (g) scMUSCLE.

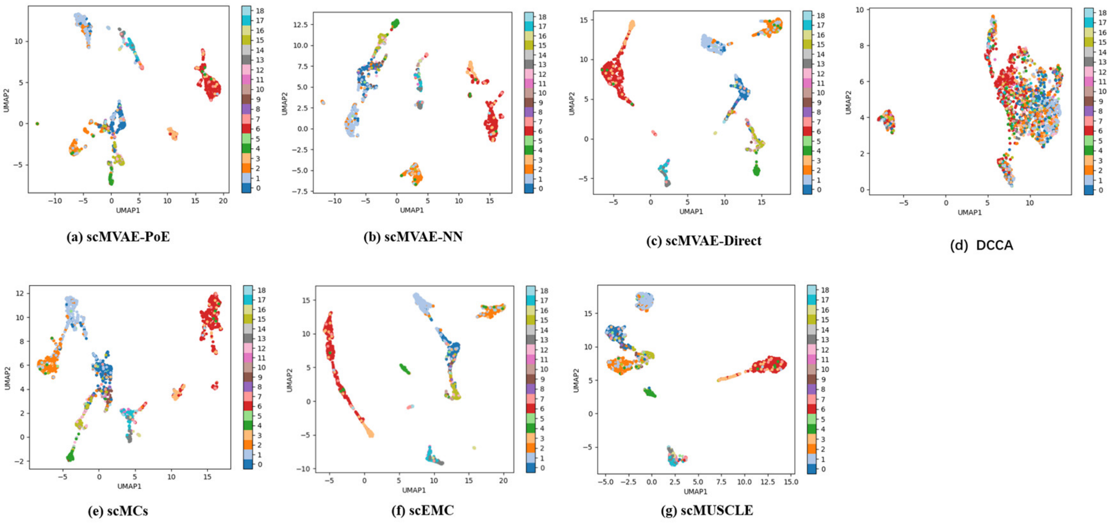

Figure S3: Cell clustering visualization of each method on PBMC. (a) scMVAE-PoE; (b)scMVAE-NN; (c)

scMVAE-Direct; (d) DCCA; (e) scMCs; (f) scEMC; (g) scMUSCLE.

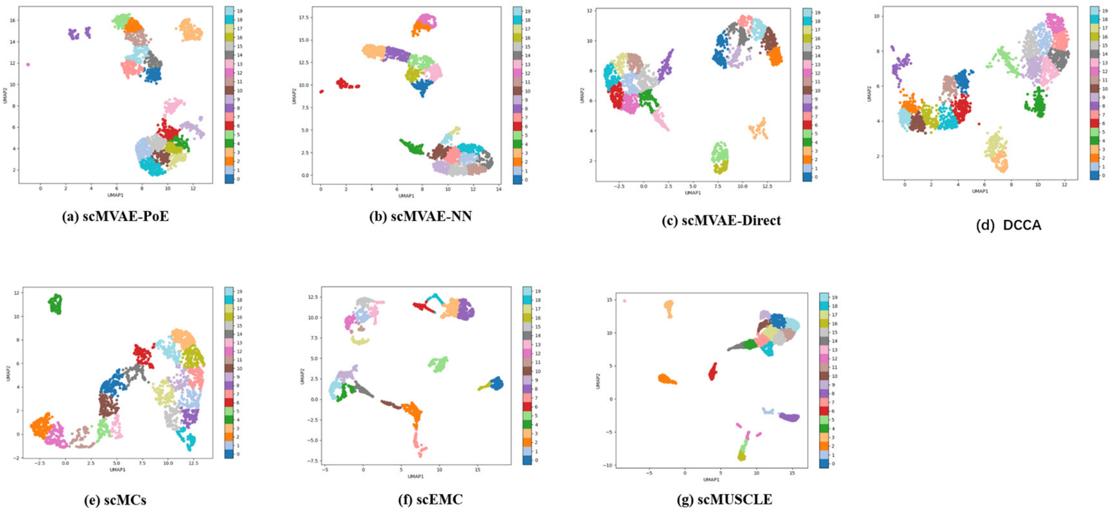

Figure S4: Cell clustering visualization of each method on h3k4me3. (a) scMVAE-PoE; (b)scMVAE-NN; (c) scMVAE-Direct; (d) DCCA; (e) scMCs; (f) scEMC; (g) scMUSCLE.

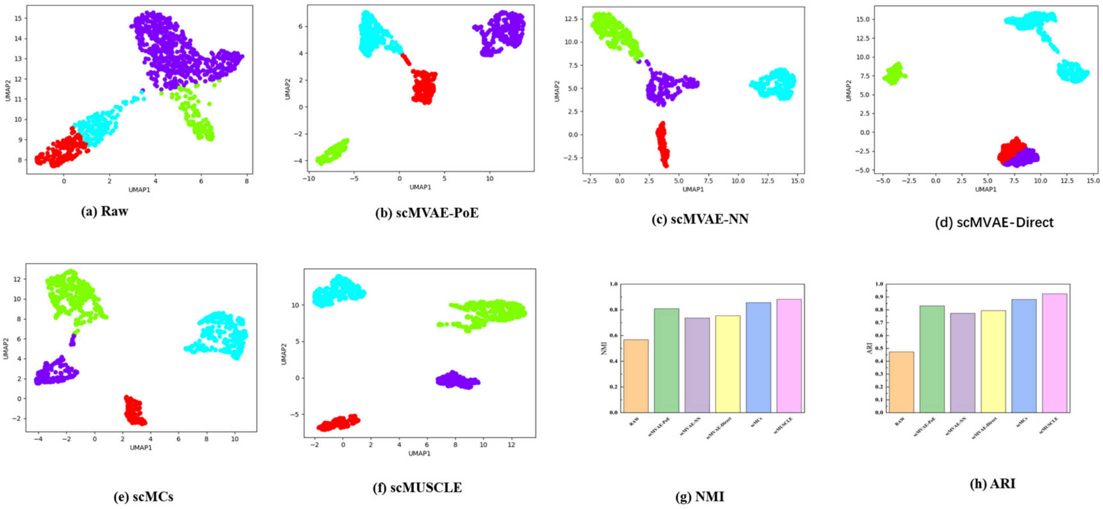

Figure S5: Cell clustering visualization of each method on raw and imputed CellMix scRNA-seq data. (a) Raw data; (b) scMVAE-PoE; (c) scMVAE-NN; (d) scMVAE-Direct; (e) scMCs; (f) scMUSCLE; (g) NMI values; (h) ARI values.

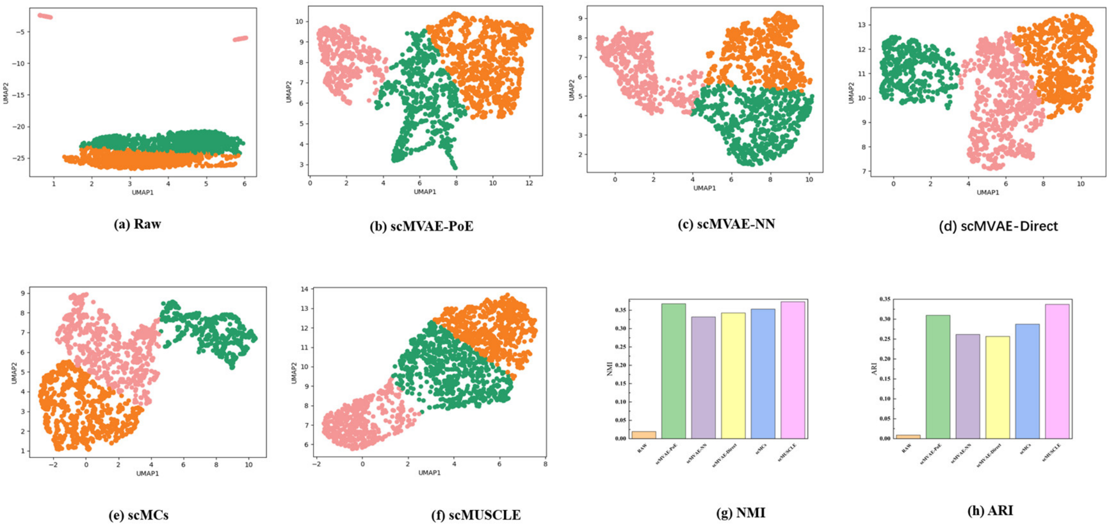

Figure S6: Cell clustering visualization of each method on raw and imputed sci-CAR scRNA-seq data. (a) Raw data; (b) scMVAE-PoE; (c) scMVAE-NN; (d) scMVAE-Direct; (e) scMCs; (f) scMUSCLE; (g) NMI values; (h) ARI values.

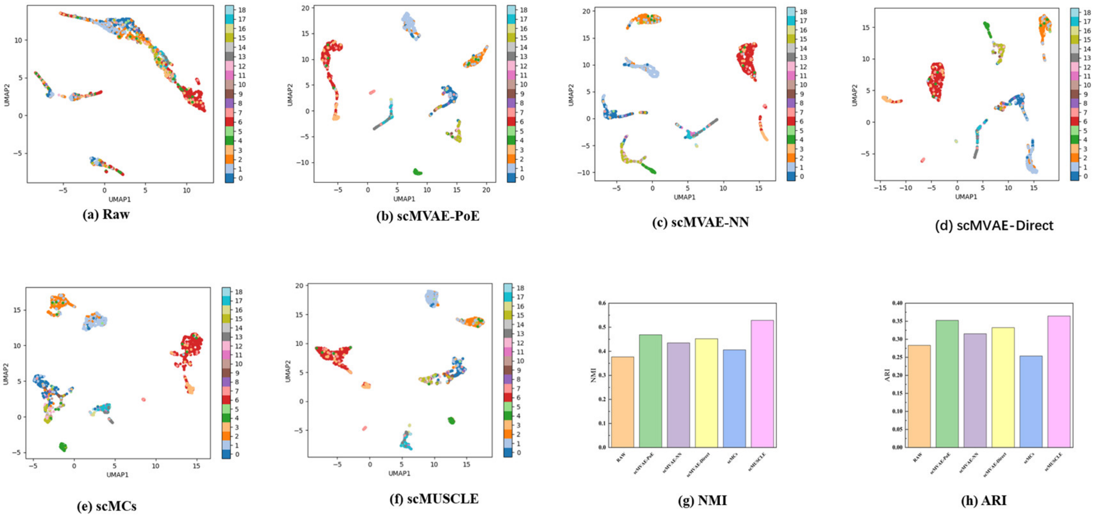

Figure S7: Cell clustering visualization of each method on raw and imputed PBMC scRNA-seq data. (a) Raw data; (b) scMVAE-PoE; (c) scMVAE-NN; (d) scMVAE-Direct; (e) scMCs; (f) scMUSCLE; (g) NMI values; (h) ARI values.

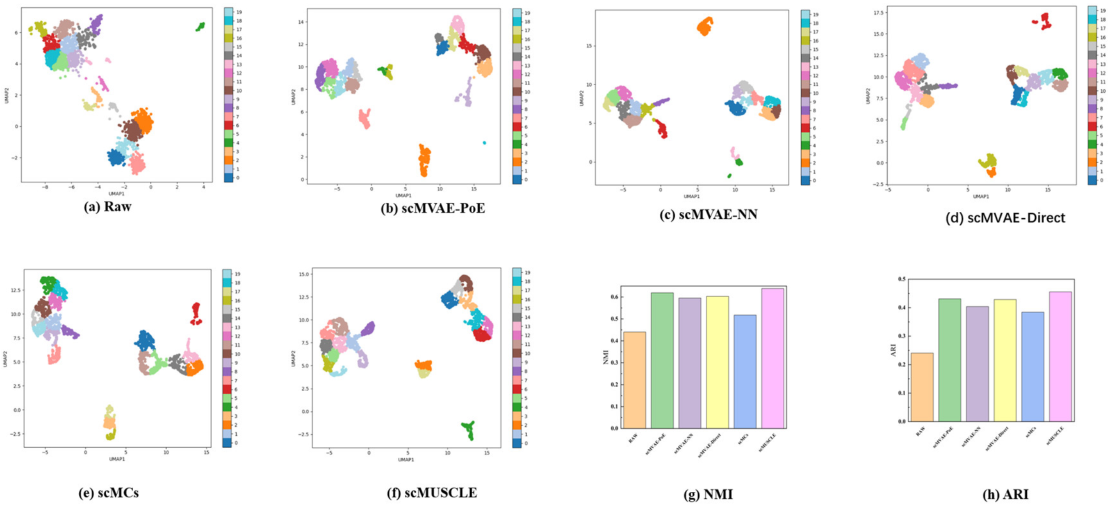

Figure S8: Cell clustering visualization of each method on raw and imputed h3k4me3 scRNA-seq data. (a) Raw data; (b) scMVAE-PoE; (c) scMVAE-NN; (d) scMVAE-Direct; (e) scMCs; (f) scMUSCLE; (g) NMI values; (h) ARI values.

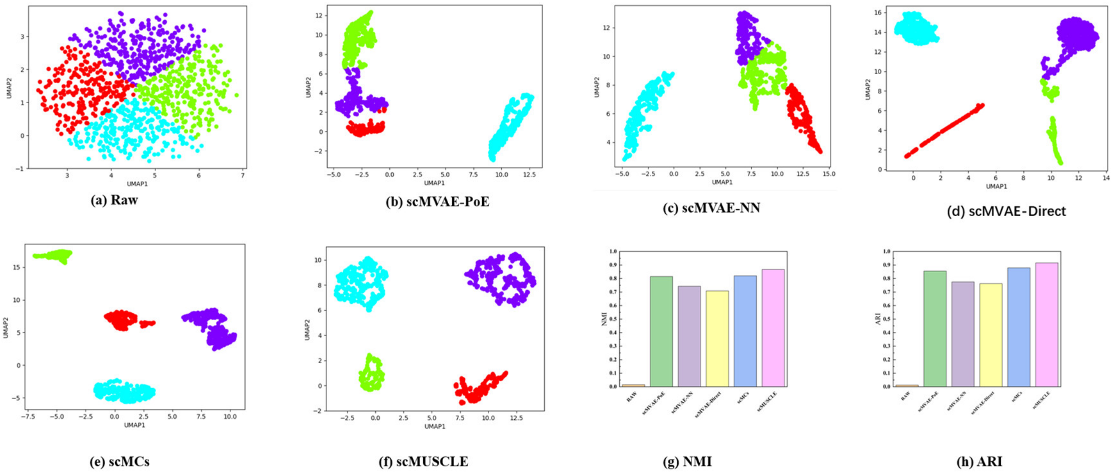

Figure S9: Cell clustering visualization of each method on raw and imputed CellMix scATAC data. (a) Raw data; (b) scMVAE-PoE; (c) scMVAE-NN; (d) scMVAE-Direct; (e) scMCs; (f) scMUSCLE; (g) NMI values; (h) ARI values.

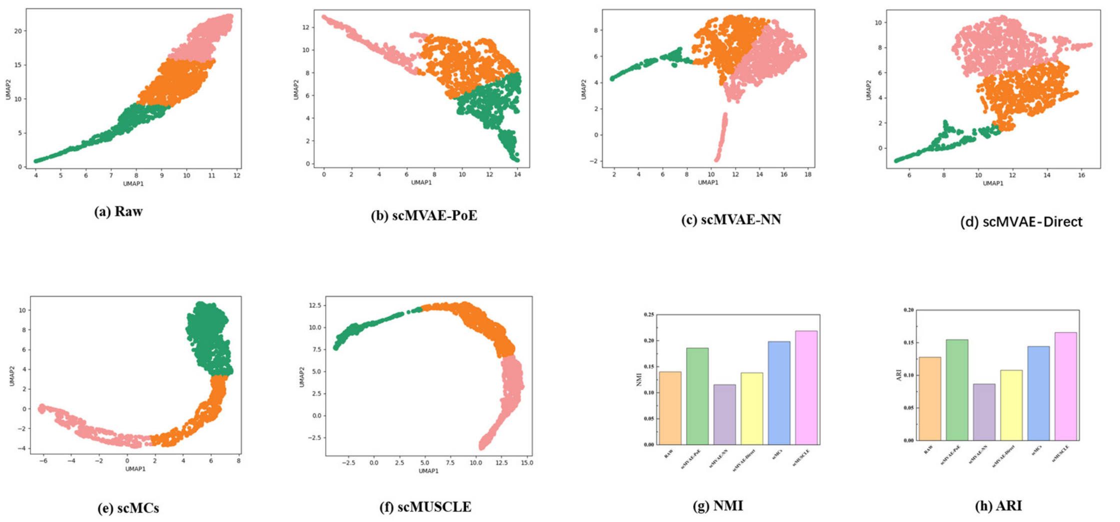

Figure S10: Cell clustering visualization of each method on raw and imputed sci-CAR scATAC data. (a) Raw data; (b) scMVAE-PoE; (c) scMVAE-NN; (d) scMVAE-Direct; (e) scMCs; (f) scMUSCLE; (g) NMI values; (h) ARI values.

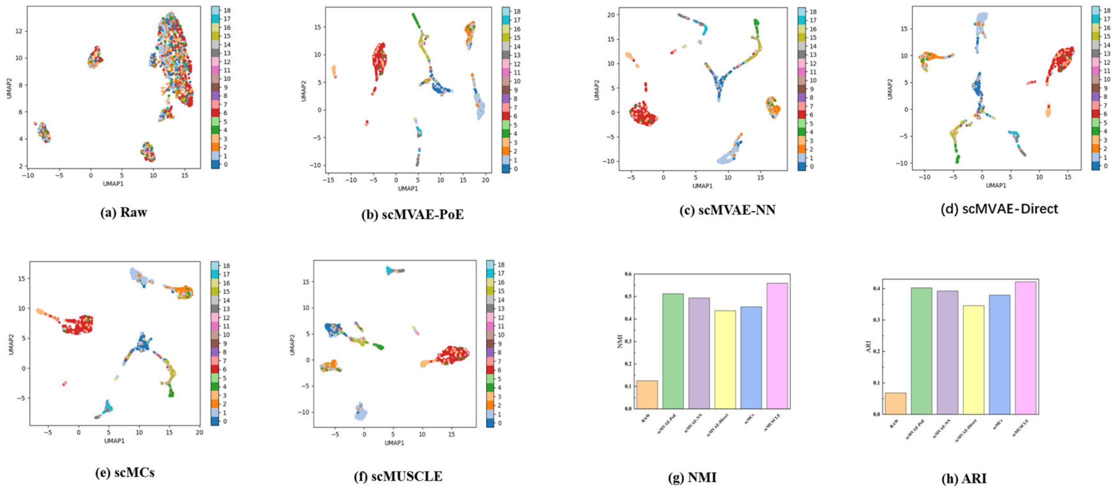

Figure S11: Cell clustering visualization of each method on raw and imputed PBMC scATAC data. (a) Raw data; (b) scMVAE-PoE; (c) scMVAE-NN; (d) scMVAE-Direct; (e) scMCs; (f) scMUSCLE; (g) NMI values; (h) ARI values.

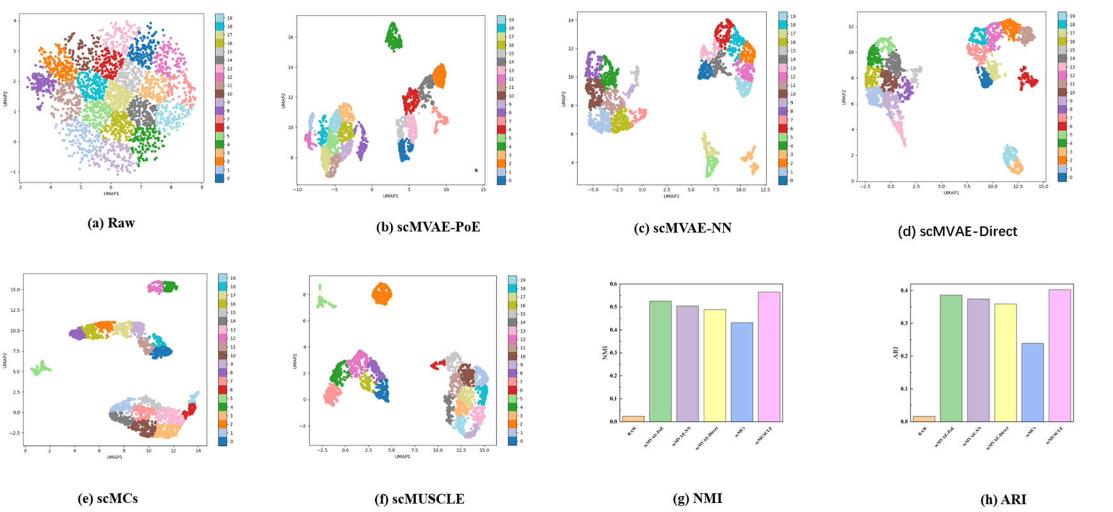

Figure S12: Cell clustering visualization of each method on raw and imputed h3k4me3 scATAC data. (a) Raw data; (b) scMVAE-PoE; (c) scMVAE-NN; (d) scMVAE-Direct; (e) scMCs; (f) scMUSCLE; (g) NMI values; (h) ARI values.

3 Ablation study

To investigate the contributions of different components in scMUSCLE, we introduced three variants: w/oCen, w/oAdaGCN, and w/oMSCL, which respectively omit centrality encoding, adaptive Graph Convolutional Neural Network (AdaGCN), and multi-subspace contrastive learning. Figure S13 shows the average NMI and ARI values of scMUSCLE and its variants. From Figure S13, we observe that scMUSCLE significantly outperforms its variants, confirming that centrality encoding, adaptive GCN, and multi-subspace contrastive learning indeed contribute to the quality of cell clustering. The results of w/oMSCL are generally the lowest, indicating the importance of multi-subspace contrastive learning in guiding cell clustering and the significance of the cross-omics shared features obtained through multi-subspace contrastive learning for consistent cell type clustering. The individual features of different omics are also important for high-quality clustering, but relying solely on individual features may lead to meaningless clustering.

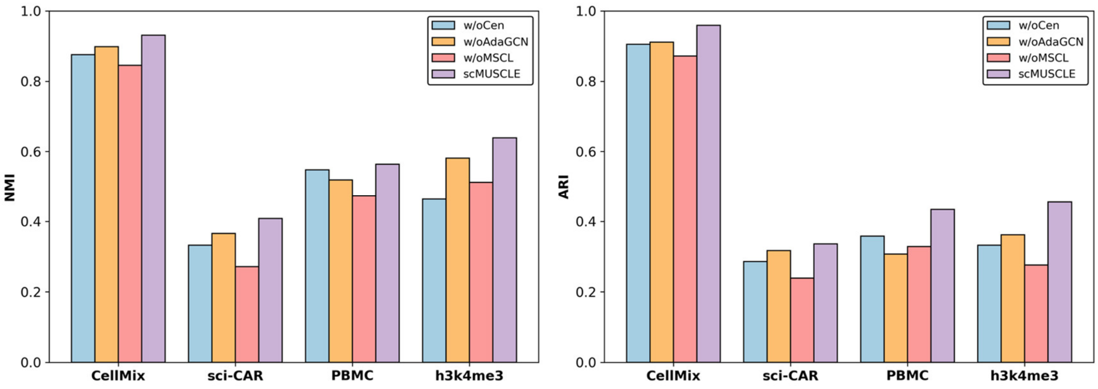

Figure S13: The performance of scMUSCLE and its variants.

4 Comparison of Clustering Performance between Single-Omics and Multi-Omics Clustering

We present the clustering results of scRNA-seq data, scATAC data, and the integration of these two omics on the CellMix, sci-CAR, PBMC, and h3k4me3 datasets in Figures S14 to S17. Figure S18 evaluates the results using two important metrics. The experimental results indicate that scRNA-seq data generally outperforms scATAC data in terms of clustering performance. The results show that multi-omics integration significantly outperforms single-omics clustering on multiple real datasets, validating the effectiveness of integrating multiple data modalities in enhancing the accuracy and robustness of cell clustering.

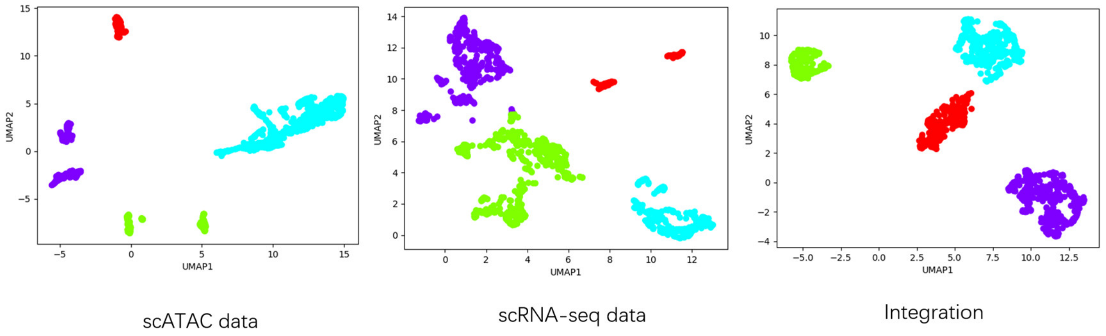

Figure S14: The figures depict the cell clustering visualizations for scATAC data, scRNA data, and the integration of these two omics on the CellMix dataset.

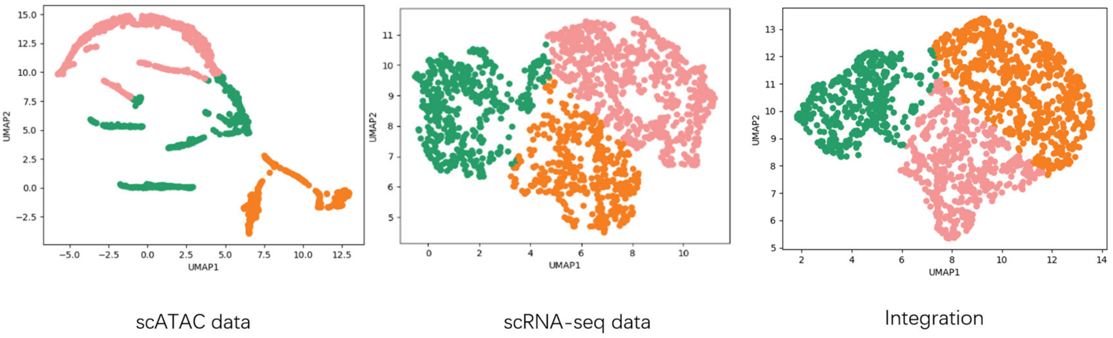

Figure S15: The figures depict the cell clustering visualizations for scATAC data, scRNA data, and the integration of these two omics on the sci-CAR dataset.

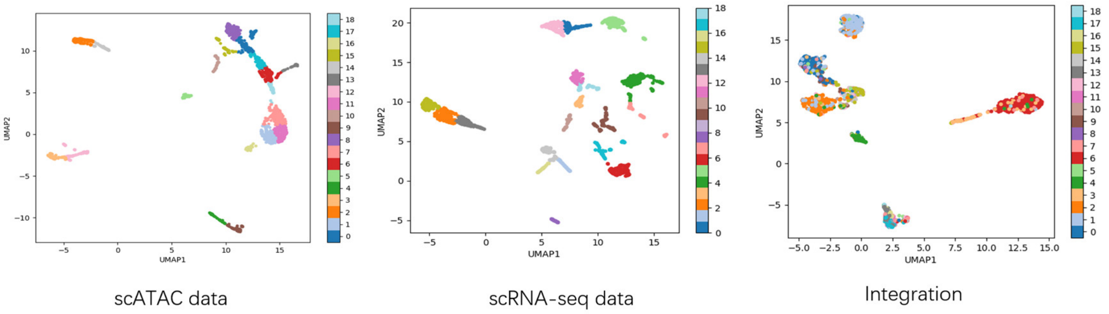

Figure S16: The figures depict the cell clustering visualizations for scATAC data, scRNA data, and the integration of these two omics on the PBMC dataset.

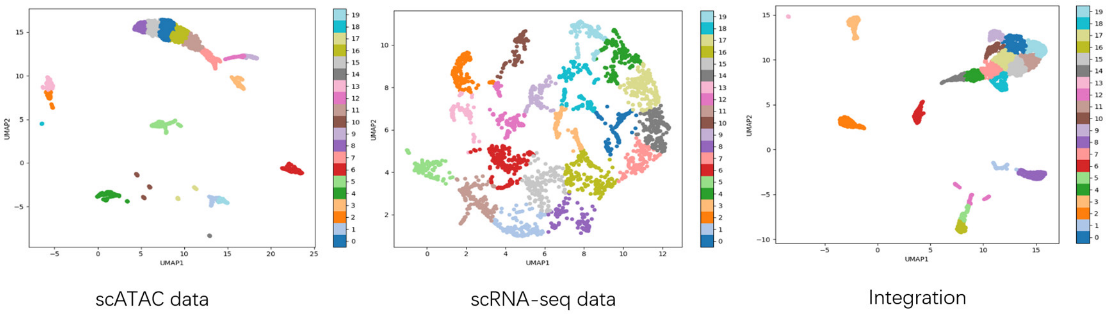

Figure S17: The figures depict the cell clustering visualizations for scATAC data, scRNA data, and the integration of these two omics on the h3k4me3 dataset.

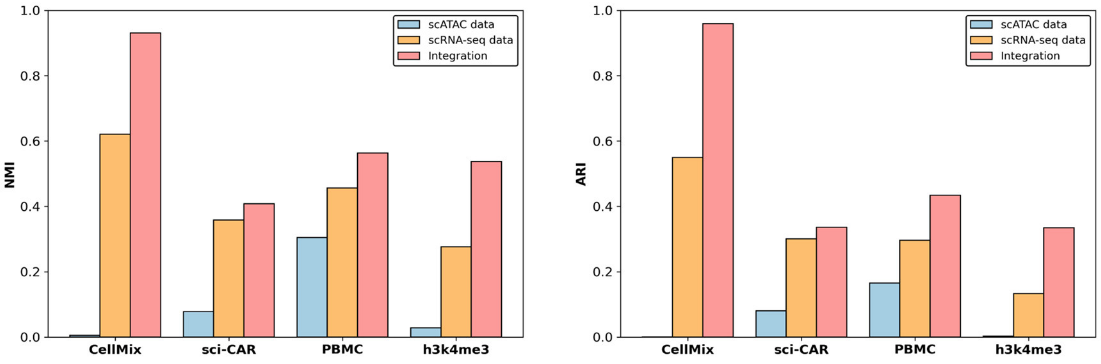

Figure S18: The figures show the NMI and ARI metrics for scATAC data, scRNA data, and the integration of these two omics on the CellMix, sci-CAR, PBMC, and h3k4me3 datasets.

We conduct different experiments to evaluate the sensitivity of scMUSCLE to different parameters combinations. The details are as follows:

4.1 Sensitivity analysis of {α1, α2}

To investigate the impact of parameters α1 and α2 on the clustering performance of scMUSCLE, we conducted experiments on these two parameters in Eq. (25). These parameters are used to balance $\mathcal{L}_{\text{Ber}}$and $\mathcal{L}_{contra}$. Through experiments on four datasets, we determined the optimal combination of α1 and α2 to optimize the clustering performance of scMUSCLE. The specific results are shown in Figures S19-S22.

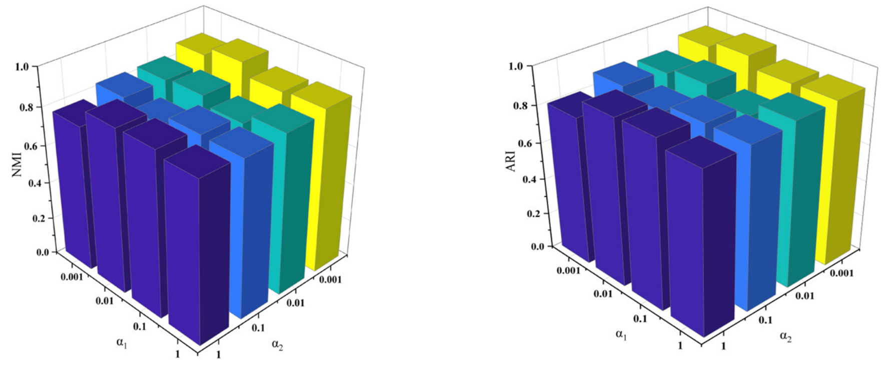

Figure S19: Sensitivity analysis of the parameters {α1, α2}on the CellMix dataset reveals that the evaluation

metrics NMI and ARI achieve their optimal values when α_1=0.01 and α_2=0.001

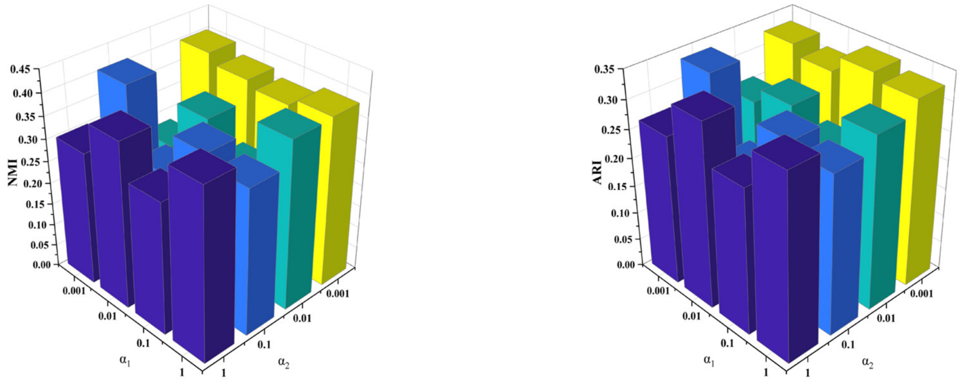

Figure S20: Sensitivity analysis of the parameters $\{\alpha_1, \alpha_2\}$ on the sci-CAR dataset reveals that the evaluation metrics NMI and ARI achieve their optimal values when α_1=0.001 and α_2=0.1

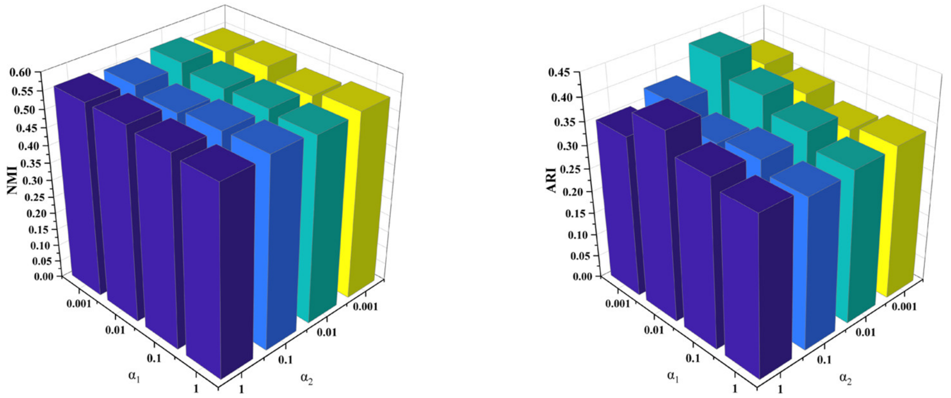

Figure S21: Sensitivity analysis of the parameters $\{\alpha_1, \alpha_2\}$ on the PBMC dataset reveals that the evaluation metrics NMI and ARI achieve their optimal values when α_1=0.001 and α_2=0.01

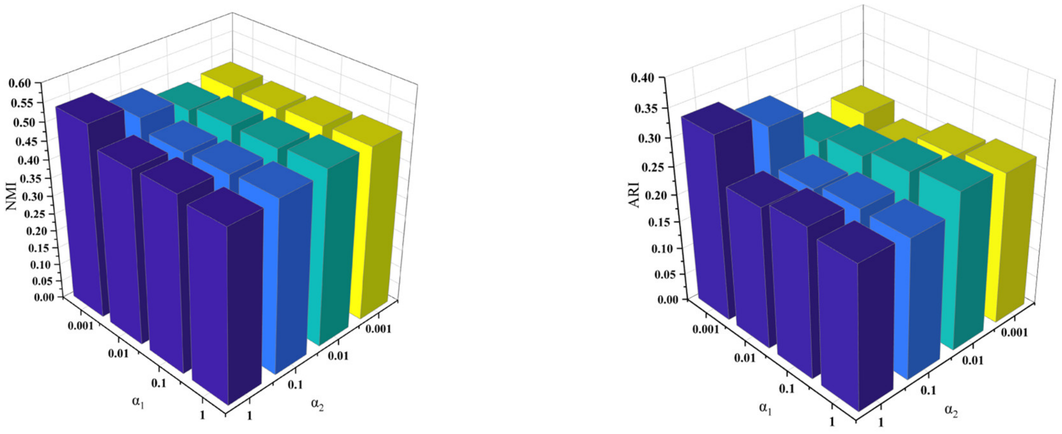

Figure S22: Sensitivity analysis of the parameters $\{\alpha_1, \alpha_2\}$ on the h3k4me3 dataset reveals that the evaluation metrics NMI and ARI achieve their optimal values when α_1=0.001 and α_2=1.

4.2 Sensitivity analysis of the number of subspaces

We also performed experiments on the number of subspaces p in multi-subspace contrastive learning, with p taking values in {1, 2, 3, 4, 5}. The clustering performance was assessed using two key metrics: NMI and ARI. It was observed that, in most cases, the optimal clustering performance was achieved when the number of subspaces p was 3.

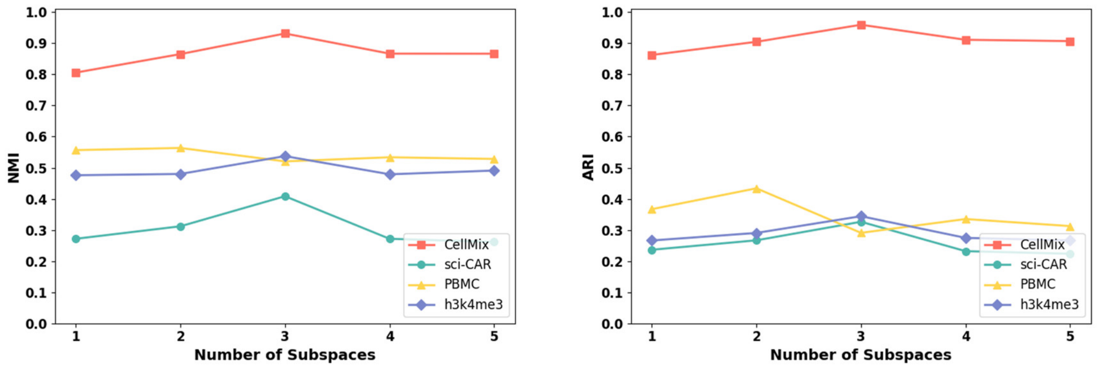

Figure S23: Sensitivity analysis of the number of subspaces on CellMix, sci-CAR, PBMC, and h3k4me3 Datasets.

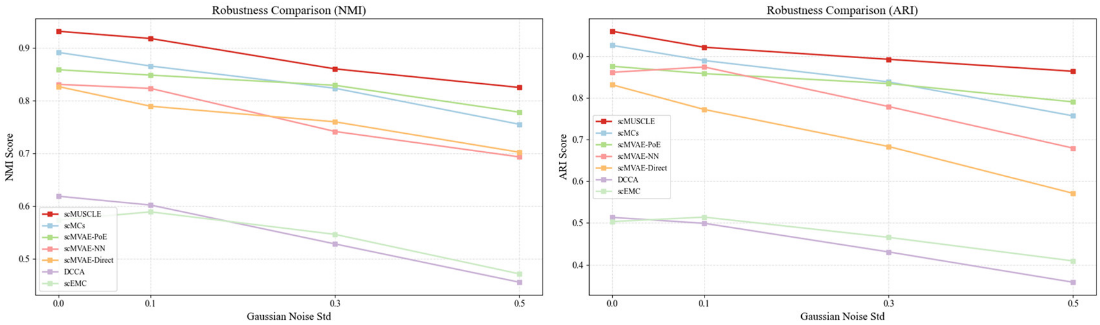

Figure S24: Robustness Evaluation under Gaussian noise perturbation on the CellMix Dataset.

5 Discussion

Multi-omics single-cell clustering methods achieve better clustering results than single-omics data, such as scRNA or scATAC data, by effectively integrating information from multiple omics (observed in Fig. 4). This integration enhances intra-cluster smoothness and compactness while enabling effective separation of inter-cluster feature information. The innovation of the proposed scMUSCLE method lies in its emphasis on the diversity extraction of low-dimensional representations of multi-omics data and the smoothness of features after integration. Specifically, the scMUSCLE method comprises two key modules: a multi-subspace contrastive learning module and an adaptive graph convolution smoothing module. These modules enable the extraction and fusion of diverse information from multiple subspaces, while establishing an adaptive graph convolution smoothing feedback mechanism based on clustering results with intra-cluster distances. This mechanism ensures the smooth consistency of multi-omics features, thereby improving the quality of single-cell clustering.

The proposed scMUSCLE method demonstrates notable robustness. When subjected to varying levels of noise in the CellMix dataset (observed in Fig. 7), the scMUSCLE method outperforms comparative methods in clustering metrics while maintaining clustering quality. Notably, under Gaussian noise with a standard deviation of 0.5, competing methods achieve Adjusted Rand Index (ARI) and Normalized Mutual Information (NMI) values below 0.8, whereas scMUSCLE consistently yielded ARI and NMI scores above this threshold. This shows scMUSCLE's superior noise tolerance in single-cell clustering tasks while delivering reliable outcomes.

The scMUSCLE method and baseline methods are evaluated by raw and imputed data clustering. When applied to unprocessed raw data, the compared methods fail to achieve effective separation between distinct single-cell clusters (observed in Fig. 3). In contrast, the scMUSCLE method demonstrates robust performance across both scRNA-seq and scATAC-seq data, yielding well-separated clusters with superior visualization outcomes.

Notably, scMUSCLE maintained its advantage when clustering imputed data, achieving the highest scores across all clustering metrics. This consistent performance underscores scMUSCLE's unique capability, which is able to extract intrinsic cellular features through multi-subspace contrastive learning, and optimize cluster structures via graph convolution smoothing. These two architecture enables the proposed scMUSCLE method to outperform alternatives in both raw data processing and imputed data analysis.

Performance with the data imputation. We carefully addressed the high sparsity and missing values inherent in single-cell multi-omics data, particularly prominent in high-dimensional sparse modalities like scATAC-seq. To tackle this, we introduced a zero-inflated negative binomial distribution in the decoder to finely model the scRNA-seq modality, capturing both discrete expression patterns and zero-inflation characteristics. For the scATAC-seq modality, we adopted a Bernoulli distribution Bernoulli distribution to align with its binarized open/closed state nature. This distribution-aware modeling in the decoder better approximates true biological statistical biological statistical properties, effectively reducing misjudgment of low-expression genes or low-accessibility regions during imputation.

Performance with the model robustness. Our approach incorporates an adaptive graph convolution module based on an intra-cluster distance feedback mechanism. By dynamically adjusting feature propagation depth according to changes in intra-cluster Euclidean distances, the module halts convolution iterations once further smoothing yields diminishing returns, thus preventing feature collapse from over-smoothing. Experiments under varying noise intensities demonstrate that scMUSCLE maintains high stability in ARI and NMI metrics, indicating its ability to adaptively adjust smoothing depth amid omics heterogeneity and sequencing noise while preserving discriminative and consistent feature representation.

Experimental results are shown in Supplementary Fig. S23 and Fig. S13. Fig. S13 presents an ablation study on multi-subspace contrastive learning where bar plots clearly indicate that this component contributes substantially to clustering performance in scMUSCLE. Fig. S23 explores the effect of varying the number of subspaces, demonstrating that as the subspace count increases, the clustering performance improves across multiple datasets. These results further validate the effectiveness of multi-subspace contrastive learning: compared to traditional shared-space methods, the proposed scMUSCLE method exhibits higher intra-class consistency and inter-class separation in multi-omics clustering tasks. This not only supports the theoretical soundness of the multi-subspace design, but also confirms its practical advantage in enhancing feature fusion and clustering outcomes.
